# How Workers Live

**Published:** 1894-06-09

**Authors:** 


### Studies in Applied Sociology.

### X.—HOW WORKERS LIVE.

Dr. Engel, the head of the Prussian Bureau of Statistics, formulated some years ago an economic law relative to the cost of living. His propositions were these:—

1. The greater the income, the smaller the relative percentage of outlay for subsistence.
2. The percentage of outlay for clothing is approximately the same whatever the income.
3. The percentage of outlay for lodging or rent, and for fuel and light, is invariably the same, whatever the income.
4. As the income increases in amount, the percentage of outlay for sundries becomes greater.

The first of these propositions is sometimes held to be a proof of the extravagance of the working class. Taking Dr. Engel's figures as examples, we find that a workman earning £60 a year spends 62 per cent. of his income on food, where a man with £250 a year spends only 50 per cent. This seems extravagant, but when we leave percentages and deal with absolute figures, the accusation falls to the ground. For 50 per cent. of £250 is £125, while 62 per cent. of £60 is only a trifle over £37; and fundamentally all human needs are the same. One has but one stomach, whether one choose for its nutriment dry bread or turtle soup. A certain quantity of food is necessary to keep the human machine in working order; all beyond that is luxury, and more or less superfluous; and taking into consideration other influences than those of the breadwinner in the house, it is not likely that it will be on food that there will be any disproportionate expenditure. Into the question of clothing vanity more readily enters, and here we find an increase. The Prussian working man averages 16 per cent. of his income for the clothing of his family, while the middle-class man spends 18 per cent. In all incomes the proportionate expenditure on fire and shelter is the same —5 and 12 per cent. respectively; but on education, care of health, and recreation of various kinds, the man with the larger income spends three times as much as his poorer brother.

The law thus formulated by Dr. Engel has been proved true not only of Prussia, but of all European countries and of the United States of America, although different ideas of the most desirable commodity cause the natives of different countries to spend different proportions of their income on the various items. Thus, taking for comparison Great Britain, Prussia, and the State of Massachusetts, we find that the English spend on subsistence 51·36 per cent. of their income, the Prussians 55 per cent., and the New Englanders 63. The expenditure on clothing is nearly the same in both European countries—18 per cent.; while in Massachusetts, it amounted in 1875 to no more than 10½ per cent. This is a little remarkable, seeing that food is cheap in America and clothing comparatively dear. We must infer, then, that things which are plentiful and therefore cheap are largely consumed, while commodities which are proportionately dear are regarded as largely unattainable, and are less indulged in. In the items of expenditure which we have classed as "sundries"—education, recreation, legal and medical expenses—we find Massachusetts most moderate, 5 per cent. of the income being the average, while the Prussian spends 10 per cent., and the Englishman 13½. It must, of course, be remembered that we are speaking of proportions, not of absolute sums. Thus while praising the comparative economy of Massachusetts in the matter of dress, we must note that, absolutely, each American family spend a fourth more than the corresponding English one—£24 to £18. But the income of the American family was £50 larger than the English. This is a fact which must always be borne in mind, and in drawing conclusions one must always look to the fact that a proportionately large expenditure may mean an absolutely small income.

Another fact, also, strikes one who reads the statistics from which these facts are drawn (the admirable report of the American Commissioners of Labour for 1892), viz., that the income of workers in America is generally larger than in Europe. The statistics of the commissioners deal with classes which are socially equal on both sides of the Atlantic, but they are by no means equal in point of income. In comparing figures we find that in the smallest incomes the European families preponderate—those from £40 to £60 a year. After that the proportions are reversed, and the progress goes steadily onward until at 800 dollars (£160) the European equivalent is almost eliminated. Taking the figures as we find them, however, we learn that in America workers pay nearly half as much again for rent as they do in Europe; for fuel only a fraction more, and for lighting little more than half as much. For food they spend, in proportion, more; but this implies no comparative lack of appreciation on the Englishman's part for his dinner; for we find that when the Briton goes to America and earns the larger American wage, his food account swells beyond that of the native. The American workman indeed is, speaking generally, more economical than any of the immigrants, who, coming from poverty, seem to be overtaken by the intoxication of plenty. For rent, the foreign workman almost invariably pays more than the native, probably because he cannot habituate himself to less accommodation than he had at home. But withal, the American is not the most provident of citizens, and the immigrants of the S nationalities, the Scotch, the Swiss, and the Swedes, more generally show a surplus of income over expenditure. It is strange that the Irish and the French Canadians, those who at home live most sparingly, become most lavish when they go to the States; and as their labour is not often of the most remunerative kind, the result is a not infrequent deficit in the exchequer. Different is it with the Germans, who preserve almost everywhere a *via media* in respect to expenditure, and who are very rarely found on the wrong side of the balance. So much for the general condition of the working classes. In future papers we propose to deal with various industries in detail.

